# Whole-Genome Sequencing of Mycobacterium tuberculosis Provides Insight into the Evolution and Genetic Composition of Drug-Resistant Tuberculosis in Belarus

**DOI:** 10.1128/JCM.02116-16

**Published:** 2017-01-25

**Authors:** Kurt R. Wollenberg, Christopher A. Desjardins, Aksana Zalutskaya, Vervara Slodovnikova, Andrew J. Oler, Mariam Quiñones, Thomas Abeel, Sinead B. Chapman, Michael Tartakovsky, Andrei Gabrielian, Sven Hoffner, Aliaksandr Skrahin, Bruce W. Birren, Alexander Rosenthal, Alena Skrahina, Ashlee M. Earl

**Affiliations:** a Office of Cyber Infrastructure & Computational Biology, National Institute of Allergy and Infectious Disease, National Institutes of Health, Bethesda, Maryland, USA; b The Broad Institute of MIT & Harvard, Cambridge, Massachusetts, USA; c Republican Scientific and Practical Centre for Pulmonology and Tuberculosis, Minsk, Belarus; d Department of Microbiology, The Public Health Agency of Sweden, Solna, Sweden; e Belarusian State Medical University, Minsk, Belarus; The Johns Hopkins University School of Medicine

**Keywords:** Mycobacterium tuberculosis, multidrug resistant, extensively drug resistant, Belarus

## Abstract

The emergence and spread of drug-resistant Mycobacterium tuberculosis (DR-TB) are critical global health issues. Eastern Europe has some of the highest incidences of DR-TB, particularly multidrug-resistant (MDR) and extensively drug-resistant (XDR) TB. To better understand the genetic composition and evolution of MDR- and XDR-TB in the region, we sequenced and analyzed the genomes of 138 M. tuberculosis isolates from 97 patients sampled between 2010 and 2013 in Minsk, Belarus. MDR and XDR-TB isolates were significantly more likely to belong to the Beijing lineage than to the Euro-American lineage, and known resistance-conferring loci accounted for the majority of phenotypic resistance to first- and second-line drugs in MDR and XDR-TB. Using a phylogenomic approach, we estimated that the majority of MDR-TB was due to the recent transmission of already-resistant M. tuberculosis strains rather than repeated *de novo* evolution of resistance within patients, while XDR-TB was acquired through both routes. Longitudinal sampling of M. tuberculosis from 34 patients with treatment failure showed that most strains persisted genetically unchanged during treatment or acquired resistance to fluoroquinolones. HIV+ patients were significantly more likely to have multiple infections over time than HIV− patients, highlighting a specific need for careful infection control in these patients. These data provide a better understanding of the genomic composition, transmission, and evolution of MDR- and XDR-TB in Belarus and will enable improved diagnostics, treatment protocols, and prognostic decision-making.

## INTRODUCTION

Despite recent reductions in tuberculosis (TB) incidence and mortality, the emergence of drug-resistant Mycobacterium tuberculosis strains, responsible for 480,000 cases of multidrug-resistant (MDR) TB disease in 2014, is a severe threat to individual patients and to effective TB control (1). MDR-TB is defined as TB resistant to at least the two major first-line drugs, rifampin and isoniazid. Extensively drug-resistant (XDR)-TB is defined as MDR-TB with additional resistances to second-line fluoroquinolones and injectable drugs (2). XDR-TB has been identified in 105 countries and represents 9.7% of MDR cases globally (2).

Eastern Europe has the highest incidence of MDR-TB worldwide (1), representing up to 20% of new cases and 60% of retreatment cases. This is especially true for countries of the former Soviet Union, such as Belarus. Despite the relatively low rate of reported TB incidence (45/100,000), the high and increasing level of MDR-TB presents a very serious challenge for TB control in Belarus. A drug resistance survey carried out in the city of Minsk in 2009 and 2010 showed the highest-ever-reported level of MDR-TB, with almost half (48%) of all smear-positive pulmonary TB patients infected with a M. tuberculosis strain presenting as MDR-TB (3). More recently, a nationwide survey revealed that the MDR-TB problem in Belarus was not localized to Minsk but was widely spread throughout the country, with both new and previously treated patients having astonishingly high rates of MDR-TB (35.3% and 76.5%, respectively) (4). Previous reports implicated specific clones, usually belonging to the Beijing lineage (5), as a primary driver of MDR-TB cases in Eastern Europe, although a survey of DR-TB in Minsk found 31% of MDR cases were caused by a specific clone within the T1 spoligotype (SIT 266) (3). XDR-TB is also a critical problem in Belarus; of all countries with ≥10 reported cases of XDR-TB in 2014, Belarus had the highest proportion (29%), followed by Latvia and Lithuania (2).

A recent study, which explored the distribution of resistance-related mutations at four specific loci associated with MDR-TB in isolates from seven countries on four continents, showed that the frequency and distribution of mutations detected varied considerably among the studied countries (5). Although Belarus had the highest prevalence of MDR-TB of all the countries, it also had the narrowest spectrum of rifampin and isoniazid resistance-related mutations compared to isolates from countries where MDR-TB was much less frequent, such as Honduras, Iran, Iraq, and Uganda. In these other countries, the distribution of rifampin and isoniazid resistance-related mutations was generally broader and more heterogeneous. The limited diversity of resistance-causing mutations for rifampin and isoniazid in Belarus suggested ongoing transmission of select MDR-TB strains rather than the importation or *de novo* evolution of new MDR variants.

To gain a more complete understanding of the genomic diversity, evolution, and transmission of MDR- and XDR-TB in Belarus, we conducted whole-genome sequencing of single M. tuberculosis isolates from 97 patients in Belarus collected between 2010 and 2013. Specifically, we selected patients representing MDR-, XDR-, and susceptible-TB cases in order to examine the genetic basis of drug resistance and the relatedness and transmission of strains among patients being treated in Belarus. We also sequenced an additional 41 isolates from 34 of these same patients who were reported to have failed treatment and who were sampled over time to investigate the evolution of M. tuberculosis within patients over the course of treatment.

## RESULTS

### Known resistance-conferring genotypes explained most phenotypic resistance to first-line drugs in MDR and XDR isolates.

To better understand the genetic basis of drug resistance in MDR- and XDR-TB in Belarus, we sequenced 97 isolates that each came from a unique patient. This set included 32 MDR, 40 XDR, and 25 susceptible isolates (see Table 1 for phenotypic drug-resistant profiles). We called variants for each isolate using the H37Rv genome as a reference (Materials and Methods), searched variants against a list of known resistance-conferring mutations (given in Table S1), and compared results to each strain's antimicrobial susceptibility testing (AST) profile for first-line drugs (rifampin, isoniazid, ethambutol, and pyrazinamide). Additionally, we identified all mutations present in the genes searched described above, regardless of whether they were known to confer resistance or not (Data Set S2). Mutations known to confer resistance to rifampin and isoniazid were highly predictive of resistance to these two drugs (Table 2). While 97% of isoniazid resistance could be explained by a single mutation, *katG* S315T, six different *rpoB* mutations, including the common S450L, combined to explain 99% of the rifampin resistance in this data set.

**TABLE 1:** Antibiotic resistance phenotype counts for each of the drugs tested

Drug	No. of phenotypically resistant	No. of phenotypically sensitive	No. not tested
First-line drugs			
Isoniazid	73	24	0
Rifampin	71	25	1
Ethambutol	69	28	0
Pyrazinamide	19	7	71
Second-line drugs			
Amikacin	39	58	0
Capreomycin	44	47	6
Kanamycin	51	46	0
Ethionamide	23	74	03
Ofloxacin	50	47	0
Cycloserine	33	57	7
Other drugs			
Streptomycin	75	22	0

**TABLE 2:** Diagnostic potential of mutations at known resistance-conferring loci^*a*^

Drug	Gene	Mutation	Frequency	PPV	Sensitivity	Specificity
Rifampin	Any		70	1	0.99	1
	*rpoB*	D435Y^*b*^^,^^*c*^	2	1	0.03	1
		H445D/L/Y^*b*^^,^^*c*^	19	1	0.27	1
		S450L/W^*b*^^,^^*c*^	49	1	0.69	1
Isoniazid	Any		72	0.99	0.97	0.96
	*katG*	S315T^*c*^	71	1	0.97	1
	*inhA*	c-15t^*c*^	26	0.96	0.34	0.96
		t-8a/c^*c*^	26	1	0.36	1
Ofloxacin	Any		43	1	0.91	1
	*gyrA*	A90V^*c*^	14	1	0.28	1
		S91P^*c*^	4	1	0.08	1
		D94A/G/H/N/Y^*c*^	25	1	0.50	1
	*gyrB*	D461N	2	1	0.04	1
		N499T	1	1	0.02	1
Kanamycin	Any		40	0.90	0.73	0.91
	*rrs*	a1400g^*c*^	31	1	0.61	1
		c1483t^*c*^	1	1	0.02	1
	*eis*	g-10a	2	0	0	0.96
		c-14t	6	0.67	0.08	0.96
Amikacin	Any		32	1	0.86	1
	*rrs*	a1400g^*c*^	31	1	079	1
		c1483t^*c*^	1	1	0.03	1
Capreomycin	Any		35	0.97	0.83	0.98
	*rrs*	a1400g^*c*^	31	1	0.72	1
		c1483t^*c*^	1	1	0.02	1
	*tlyA*	LOF	3	0.67	0.05	0.98
Streptomycin	Any		70	1	0.93	1
	*rpsL*	K43R	41	1	0.55	1
		K88R	3	1	0.04	1
	*rrs*	a513c	19	1	0.25	1
		a516t	6	1	0.08	1
	*gidB*	LOF	2	1	0.03	1
Pyrazinamide	Any		3	1	0.16	1
	*pncA*	LOF	3	1	0.16	1
Ethambutol	Any		63	0.98	0.90	0.96
	*embB*	M306I/V^*c*^	38	0.97	0.53	0.96
		G406D	1	1	0.01	1
		Q497R	24	1	0.34	1
Ethionamide	Any		65	0.35	1	0.42
	*ethA*	LOF	47	0.36	0.74	0.59
	*inhA*	c-15t^*c*^	26	0.50	0.57	0.82
		t-8a/c^*c*^	26	0.23	0.26	0.73

a Isolates were included only if they had both phenotypic and genotypic resistance predictions. For each mutation, we show frequency of the mutation in the data set, positive predictive value (PPV), sensitivity, and specificity for predicting phenotypic resistance. Mutations are listed as either specific changes or as any loss-of-function (LOF) mutation, including nonsense mutations and frameshifts.

b Mutation detectable by GeneXpert MTB/RIF.

c Mutation detectable by MTBDR*plus* version 2.0 or MTBDR*sl* version 1.0.

The sensitivity for predicting ethambutol resistance was slightly lower (90%), with the primary resistance-conferring mutations in *embB* being M306I, M306V, and Q497R (Table 2). Of the seven isolates for which known ethambutol resistance-conferring mutations could not explain phenotypic resistance, two isolates carried other polymorphisms in *embB* that were found only within ethambutol-resistant strains (N296H and D354A; Data Set S2). Although these mutations were not included in our list, both mutations were recently implicated in ethambutol resistance in a study from France (6). Mutations known to confer resistance to pyrazinamide had the lowest sensitivity for predicting phenotypic resistance to this drug; only 16% of resistance could be explained when *pncA* loss-of-function mutations were included. However, when all 10 nonsynonymous mutations in *pncA* present in pyrazinamide-resistant isolates were considered, the sensitivity and specificity for detecting resistance rose to 100% (Table 3 and Data Set S2). All of these mutations have been previously implicated in pyrazinamide resistance (http://www.moleculartb.org/gb/pdf/doc/Moltb-tableau-pncA-17_12_14.pdf), with the exception of *pncA* L151W, which was present in only a single isolate.

**TABLE 3:** Nonsynonymous mutations in *pncA* and *alr* improve diagnosis of resistance to pyrazinamide and cycloserine, respectively^*a*^

Drug	Gene	Mutation	Frequency	PPV	Sensitivity	Specificity
Pyrazinamide	Any		19	1	1	1
	*pncA*	LOF	3	1	0.16	1
		D8N	1	1	0.05	1
		Q10R	1	1	0.05	1
		D12A	1	1	0.05	1
		V21A	1	1	0.05	1
		D49G/Y	5	1	0.26	1
		H57D	1	1	0.05	1
		W68G/R	5	1	0.26	1
		L151W	1	1	0.05	1
Cycloserine			15	0.73	0.33	0.93
	*alr*	L89R	15	0.73	0.33	0.93

a Isolates were included only if they had both phenotypic and genotypic resistance predictions. For each mutation, we show the frequency of the mutation in the data set, positive predictive value (PPV), sensitivity, and specificity for predicting phenotypic resistance. Mutations are either listed as specific changes or as any loss-of-function (LOF) mutation, including nonsense mutations and frameshifts.

While most strains had resistance-conferring mutations that matched their first-line AST profiles, isolate XTB13-299 was phenotypically MDR but was genetically susceptible based on our list of known mutations. Examination of sequencing reads for XTB13-299 revealed low-frequency polymorphisms at known resistance-conferring sites within *rpoB* (5 of 237 aligned reads contained the *rpoB* S450W variant), the *inhA* promoter (2 of 266 aligned reads contained the *inhA* c-15t variant), and *embB* (1 of 229 aligned reads contained the *embB* M306I variant). These low-frequency mutations suggest that this patient might have been infected with a mixed population of M. tuberculosis, with the minority strain leading to the MDR phenotype.

We also evaluated strains for mutations in *rpoA* and *rpoC*, mutant forms that are known to compensate for reductions in fitness caused by *rpoB* mutations (7, 8). We identified 11 unique single nucleotide polymorphisms (SNPs) in *rpoC* and 2 SNPs in *rpoA* present only in rifampin-resistant isolates (Data Set S2). *rpoC* I491T was the most prevalent SNP cooccurring with the *rpoB* S450L mutation and was previously predicted to compensate for the *rpoB* S450L (Escherichia coli S531L) mutation (8). Similarly, most other potential compensatory mutations seen here (Data Set S2) were previously reported (7–11). The exceptions were *rpoC* E518D, E757G, and A1213E and *rpoA* G31S variants, which may represent novel compensatory sites in these genes.

### Known resistance-conferring genotypes explained most phenotypic resistance to second-line fluoroquinolones and injectables in MDR and XDR isolates.

Second-line treatment of TB for the patient cohort included an aminoglycoside/cyclic peptide, a fluoroquinolone, ethionamide, cycloserine, and *para*-aminosalicylic acid. Of all second-line drugs, fluoroquinolone (ofloxacin) resistance was most often (91%; Table 2) successfully predicted by known resistance-conferring mutations from our list. Seven different resistance-conferring polymorphisms were identified in *gyrA*, and two were identified in *gyrB*, the most common being *gyrA* A90V, D94A, and D94G. Known resistance mutations had a lower sensitivity for predicting resistance to injectables (aminoglycosides and cyclic peptides), ranging from 73% for kanamycin to 83% for capreomycin and 86% for amikacin. The most common mutation was *rrs* a1400g, which provides cross-resistance to all three aforementioned aminoglycosides and cyclic peptides. Some strains also had kanamycin-specific (*eis* promoter) or capreomycin-specific (*tlyA* loss of function) mutations.

Ethionamide resistance was poorly predicted in this data set; while the sensitivity was 100%, the specificity was only 42%. Loss-of-function mutations in *ethA* had a particularly low specificity of 59%. This effect was not driven by any single mutation but rather poor specificity across a wide range of loss-of-function mutations. No cycloserine resistance was initially predicted, as the only mutation on our list, loss of function of *ald*, was absent in all samples. However, 15 cycloserine-resistant strains harbored an *alr* L89R, a mutation that was previously identified in cycloserine-resistant clinical isolates from patients treated in South Africa (12). Although not yet experimentally validated, the addition of this mutation to our list increased sensitivity in this data set to 33%, with 100% specificity (Table 3).

### Phylogenetic analysis suggests uncontrolled transmission of MDR-TB in Belarus.

**FIG 1:**
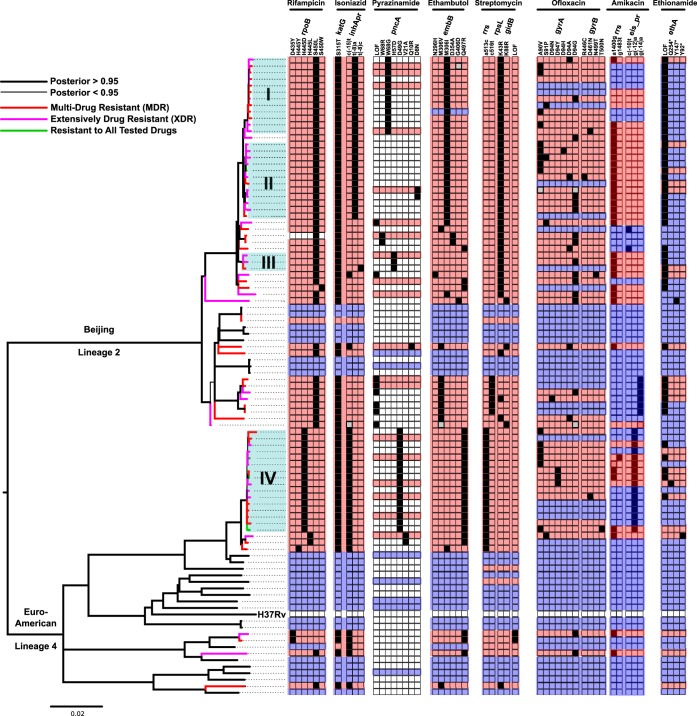
Phylogeny and drug resistance diversity among M. tuberculosis isolated from patients in Belarus from 2010 to 2013. The phylogenetic tree was created using MrBayes and rooted between the Beijing and Euro-American lineages. Thicker branches indicate a posterior probability support of >0.95, and strains phenotyped as MDR, XDR, or resistant to all tested drugs are indicated by color at the terminal branches of the tree. Four closely related clades of MDR and XDR isolates are labeled (I, II, III, and IV). The results of computational spoligotyping (see Materials and Methods) are also shown along representative branches and nodes. A heat map is shown that illustrates, for each strain, the presence of known drug resistance-conferring variants detected in isolate genomes. Clear cells in this heatmap indicate that no known drug resistance variant was detected; black cells indicate that a high-confidence drug resistance variant was detected, and gray cells indicate that there was ambiguity in the variant call made at those positions. A red background indicates the isolate was phenotypically resistant, a blue background indicates the isolate was phenotypically sensitive, and a white background indicates the isolate did not undergo AST for that drug.

To better understand whether the spread of MDR- and XDR-TB in Belarus is driven by person-to-person transmission or *de novo* evolution of resistance within individual patients, we estimated a Bayesian phylogeny from SNPs predicted in the 97 isolates. The phylogeny revealed that all isolates fell into either the Beijing lineage (lineage 2) or the Euro-American lineage (lineage 4; Fig. 1). MDR isolates were significantly more likely to belong to the Beijing lineage than the Euro-American lineage (*P* < 0.03, Fisher's exact test), although XDR isolates did not show such a bias (*P* < 0.68; Fisher's exact test). The phylogenetic analysis also demonstrated that many MDR and XDR isolates clustered into four shallow well-supported (posterior probability >0.95) clades containing closely related strains (mean pairwise distance, 11 to 15 SNPs; Fig. 1, clades I, II, III, and IV). This close similarity among drug-resistant M. tuberculosis strains was comparable to the level of variation recently observed in a single patient in which drug resistance evolved over the course of treatment (13), suggesting an important role for the transmission of MDR- and XDR-TB among patients included in this study.

To quantify the relative importance of person-to-person transmission and *de novo* evolution of resistance within individual patients for each drug, we used parsimony and the data presented in Fig. 1 to estimate the number of times genotypic resistance evolved in isolates with AST results. For each drug, we divided this estimate by the total number of phenotypically resistant isolates to determine the fraction of resistance acquired through *de novo* evolution (Table 4). Our analysis revealed that most of the resistance to isoniazid and rifampin was acquired by patients through transmission (90% and 87%, respectively). This was not the case for second-line drugs. While we predicted that the majority (74%) of amikacin-resistant TB was transmitted to these patients, the majority (54%) of ofloxacin resistance was predicted to have arisen *de novo* within this population, suggesting a role for both transmission and intrahost evolution in the rising incidence of XDR-TB in Belarus.

**TABLE 4:** Fraction of drug resistance acquired through person-to-person transmission versus *de novo* evolution of resistance within individual patients for each drug^*a*^

Drug	No. of acquisitions	Estimated % *de novo*	Estimated % transmission
First-line drugs			
Isoniazid	7	10	90
Rifampin	9	13	87
Ethambutol	15	22	78
Pyrazinamide	9	47	53
Second-line drugs			
Amikacin	10	26	74
Ethionamide	9	39	61
Ofloxacin	27	54	46
Other drugs			
Streptomycin	10	13	87

a Parsimony was used to estimate the number of times genotypic resistance was gained in isolates with AST results. This number was divided by the total number of phenotypically resistant isolates to estimate the fraction of resistance to each drug acquired through *de novo* evolution, while the remaining fraction was assigned to acquisition from person-to-person transmission.

While streptomycin was not given to patients as either a first- or second-line therapy during the course of treatment for this study, all MDR and XDR isolates harbored resistance to streptomycin through a number of known resistance-conferring mutations in *rpsL*, *rrs*, and *gidB* (Table 2). While we estimated that most streptomycin resistance was acquired through person-to-person transmission (Table 4), parsimony still predicted 10 independent instances of streptomycin resistance, suggesting that strains acquired this resistance through exposure to streptomycin through treatment prior to this study. As streptomycin was widely used for the treatment of TB in Belarus up to 2012, exposure of circulating M. tuberculosis strains to this drug could have frequently occurred.

### Longitudinal samples highlight intrahost evolution and multiple infections.

**FIG 2:**
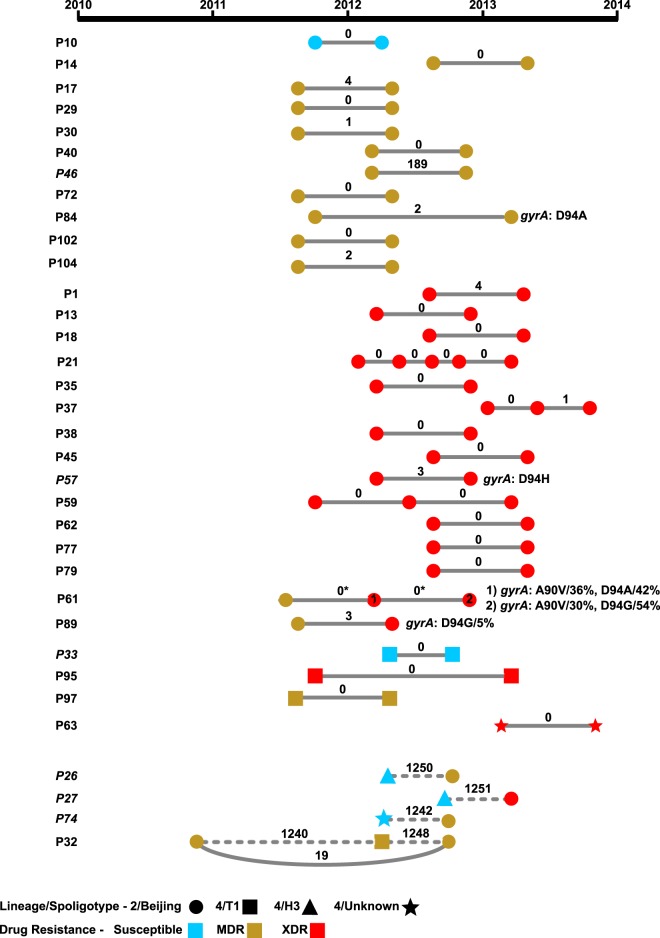
Genetic diversity and drug resistance in serial M. tuberculosis isolates. Individual samples from the same patient are shown as nodes connected by lines. Spoligotypes are indicated by node shape, and same-patient samples from the same or different spoligotype are connected by solid and dashed lines, respectively. Node colors correspond to phenotypic drug resistance. Samples are ordered by spoligotype and by patient number within each spoligotype, and mixed-spoligotype samples are presented last in the figure. The number of SNPs that differ between samples is indicated on the connecting lines. SNPs involving changes that are predicted to impact drug resistance are listed; patient 57 was initially fluoroquinolone resistant yet acquired a second fluoroquinolone resistance-conferring SNP. Percentages indicate prevalence. Asterisks indicate cases where no SNPs were identified between samples, but evaluation of read data indicated the presence of low-frequency variants conferring drug resistance. HIV+ patients are indicated by italics.

In addition to understanding how MDR- and XDR-TB are evolving and spreading among patients within Belarus, it is also critical to understand the genomic context of a TB infection when treatment fails. We, therefore, sequenced an additional 41 isolates from the original 97 isolates among 34 patients who maintained positive sputum cultures over time, i.e., their infections persisted despite treatment. These included 29 patients with TB that persisted but did not gain additional drug resistances over time and 5 patients with TB that became more resistant over time (Fig. 2).

When we identified SNPs that differed across same-patient isolates, we observed that most patients (29 of 34) maintained strains that either remained genetically unchanged (0 SNPs) or showed a pattern consistent with intrahost evolution (1 to 4 SNPs) (Fig. 2). In three of these cases, a new phenotypic resistance was acquired, and in all of those cases, the resistance gained was to ofloxacin. However, only one case of phenotypic ofloxacin could be explained using our list of known resistance-conferring mutations (patient 84; Fig. 2). However, examination of sequencing reads revealed resistant subpopulations in the other two patients (61 and 89). In the sample from patient 89, the *gyrA* D94G mutation was detected at 5% frequency in the sequencing reads. In the sample from patient 61, known resistant alleles, *gyrA* A90V and D94A, comprised 36% and 42% of the sequencing reads, respectively, aligning across these sites. A third sample taken at least 6 months later from patient 61 showed approximately the same level of heterogeneity at these two positions (Fig. 2) indicating that ofloxacin-resistant and -susceptible subpopulations can coexist in a patient for many months. Daily fluoroquinolone was part of each MDR patient's treatment over the period when these samples were collected (Data Set S1).

Five patients showed a clear pattern of multiple infections rather than evidence for intrahost evolution of the same strain, having >150 SNP differences between isolates sampled at different time points (patients 26, 27, 32, 46, and 74; Fig. 2). In four patients, longitudinal isolates differed by over 800 SNPs and were part of distinct lineages, while the remaining patient isolates belonged to the Beijing lineage and differed by 189 SNPs. In three of the five cases, the earlier isolates were more drug susceptible than the later isolates, which were genotypically predicted to survive the patient's first-line treatment (treatment details are given in Data Set S1). In one of the remaining two cases with three time points, an MDR strain was present at the first and third time points, while the second sample was unrelated but also MDR, suggesting this patient may have been simultaneously harboring multiple MDR strains. In the final remaining case, the patient harbored two XDR strains at different time points. Strikingly, of the five patients showing a pattern consistent with multiple infections, four patients were HIV+ (of 5 total HIV+ patients in this study), representing a significantly greater likelihood of multiple infections in HIV+ patients than in patients not infected with HIV (*P* < 0.001, Fisher's exact test).

## DISCUSSION

To better understand the genetic basis and transmission of MDR- and XDR-TB in Belarus, we sequenced and analyzed 97 isolates distributed across MDR, XDR, and susceptible phenotypes. Known resistance-conferring loci accounted for the majority of phenotypic resistance to first- and second-line drugs for MDR- and XDR-TB isolates in our study. Phylogenetic analysis suggested that most MDR-TB was due to the transmission of already-resistant M. tuberculosis strains rather than *de novo* evolution of resistance within patients, while XDR-TB was acquired through both routes. Sequencing of an additional 41 longitudinally sampled isolates from 34 patients with treatment failure showed that most strains persisted genetically unchanged during treatment or acquired resistance to fluoroquinolones in progression toward an XDR status.

The vast majority of phenotypic resistance to first-line drugs (rifampin, isoniazid, and ethambutol) in MDR- and XDR-TB was well predicted by known resistance-conferring mutations. Additionally, while known resistance-conferring mutations performed well in predicting resistance to second-line fluoroquinolones in MDR- and XDR-TB, they overpredicted resistance to ethionamide and underpredicted resistance to aminoglycosides, cycloserine, and pyrazinamide. Pyrazinamide resistance detection could be improved by the incorporation of a number of rare nonsynonymous changes, which has been noted previously (14). It should also be noted that sampling focused on MDR- and XDR-TB, forms of TB that we considered the highest priority for diagnosis, treatment, and infection control, and not the relative prevalence of individual phenotypic drug resistances in Belarus. Therefore, our sensitivity and specificity results should be interpreted only for MDR- and XDR-TB.

Our phylogenetic analysis supported the hypothesis that MDR-TB in Belarus was primarily being acquired through person-to-person transmission. Additionally, the small level of genetic diversity found within a number of MDR clades in this data set was consistent with levels found across time course isolates from a single patient (13). A previous study of DR-TB detected limited variation at drug resistance-conferring loci in samples from Belarus compared to other countries and hypothesized that this was due to a greater proportion of transmitted resistant TB (as opposed to *de novo* evolution of resistance) (5). While we also conclude there is extensive transmission of drug-resistant TB in Belarus, we observed a much greater variety of resistance mutations among sequenced strains than previously reported. This is likely due to increased sample size, which allowed us to capture less-prevalent mutations. However, we acknowledge that our estimation of the relative role of transmission and *de novo* acquisition of resistance is an estimate, and it could be biased if the selected samples were not representative of all circulating strains. In particular, estimates could be biased toward transmission if large hospital outbreaks of MDR-TB were oversampled.

Unlike MDR-TB, the acquisition of XDR-TB appeared to be driven by both patient-to-patient transmission and *de novo* evolution of resistance. *De novo* evolution of resistance was particularly true for fluoroquinolones, as we observed repeated acquisition of resistance in both the cross-sectional and longitudinal data sets. Strains in three patients evolved fluoroquinolone resistance over the course of treatment, two of which were present as a mixed community of susceptible and resistant isolates. Heteroresistance to fluoroquinolones was also seen in one additional patient in the 97-isolate set and has been previously noted in studies of fluoroquinolone-resistant TB (15, 16). A previous study from Russia also noted that fluoroquinolone resistance appeared to be independently acquired more frequently than resistances to other drugs (17). These patterns suggest that resistance to fluoroquinolones may have a greater fitness cost than resistance to other drugs, making it more difficult for fluoroquinolone resistance-conferring mutations to become fixed in intra- and interhost populations. Furthermore, this heteroresistance can lead to discrepancies between results obtained from AST and genome sequencing.

Analysis of time course data of cases of treatment failure revealed that most strains, despite appropriate treatment, remained stable in patients; 85% of the 34 treatment failure cases had phenotypic and genomic susceptibility to at least one drug they were being given. This included two fully drug-susceptible strains, indicating that treatment failure is not limited to drug-resistant cases. The limited *de novo* evolution of resistance in this patient population (aside from fluoroquinolone resistance acquisition) is in contrast to previous studies, where extensive intrahost evolution of drug resistance was well documented (13, 18). While we documented cases of mixed infection of both treatment-susceptible and -resistant strains, the fact that susceptible strains were not eliminated by treatment indicates possible problems with treatment adherence, absorption of the drugs in the gastrointestinal tract, or drug quality, as well as host immunosuppression.

Despite most strains persisting unchanged in patients, three patients had drug-susceptible strains isolated at earlier time points convert to unrelated drug-resistant strains at later time points. Our primary hypothesis is the patient was cured of the susceptible TB but was later reinfected with a more resistant strain. However, mixed infections could have been present and gone undetected due to limited sampling, as hypothesized for the one patient harboring two drug-resistant strains at multiple time points, and we cannot rule out the possibility that contamination or laboratory error resulted in mixed samples. Furthermore, this potential recolonization by drug-resistant strains was particularly apparent in HIV+ patients, in line with a recent study of TB in South America that showed that HIV positivity increased the likelihood of contracting tuberculosis (19). Therefore, it may be particularly important that immunocompromised TB patients limit exposure to drug-resistant cases of TB, particularly within health care settings.

Overall, these data provide insight into the genomic composition and modes of acquisition of MDR- and XDR-TB, critical health care problems in Belarus. Improved infection control will be of critical importance to stop the growing spread of MDR-TB and reinfection of particularly susceptible populations. However, these data also suggest that *de novo* evolution of XDR-TB is still frequently occurring and must be accounted for in strategies to control drug-resistant TB in Belarus.

## MATERIALS AND METHODS

### Sample acquisition and selection.

Initial samples from patients in Minsk, Belarus, from 2010 to 2013, were taken at the time of TB diagnosis and treatment initiation, and the resulting cultured isolates were stored at −70°C at the Belarusian National Reference Laboratory (NRL) in Minsk. Follow-up samples were taken from patients who maintained a positive sputum culture over time, i.e., failed treatment, every 5 to 6 months postdiagnosis for patients with susceptible TB and 6 months to 1 year postdiagnosis for patients with MDR- or XDR-TB, although in a small number of cases, samples were taken more frequently. Sampling time points, HIV status, and drug therapy frequency and dosage are presented in Data Set S1. An initial set of 97 isolates in which each isolate came from a unique patient was selected to provide a balance among drug resistance profiles; this set included 32 MDR, 40 XDR, and 25 additional isolates, including 22 isolates susceptible to all tested drugs, 2 isolates resistant to only streptomycin, and 1 isolate resistant to isoniazid and streptomycin. For simplicity, we refer to these 25 isolates as “susceptible.” From patients who maintained a positive sputum culture over time, i.e., failed treatment, an additional 41 isolates were selected, resulting in a second set of 75 isolates from longitudinally sampled patients. In total, 138 isolates were sequenced, with 34 isolates overlapping both sets. For all longitudinally sampled patients, the sample with the latest date was used in the 97-isolate set.

### Antimicrobial susceptibility testing.

The *in vitro* antimicrobial susceptibility testing (AST) was carried out at the NRL in Minsk, which was externally quality assured by the WHO Supranational Reference Laboratory at the Public Health Agency of Sweden (PHAS) in Stockholm. AST was done with the absolute concentration method on Lowenstein-Jensen (LJ) medium with the following critical concentrations: 1 mg/liter isoniazid (INH), 40 mg/liter rifampin (RIF), 2 mg/liter ethambutol (EMB), 4 mg/liter streptomycin (STR), 30 mg/liter kanamycin (KAN), 30 mg/liter amikacin (AMK), 40 mg/liter capreomycin (CM), 4 mg/liter ofloxacin (OFX), 40 mg/liter ethionamide (ETH), 30 mg/liter cycloserine (CS), and 1 mg/liter *para*-aminosalicylic acid (PAS). Pyrazinamide (PZA) testing was performed using Bactec MGIT PZA kits (BD) at a concentration of 100 μg/ml. AST was conducted on the initial isolate and in most cases was only done once. However, isolates that were found to have a resistant genotype during the study and a corresponding susceptible phenotype were retested for that drug and the data were corrected if the phenotypic retesting differed from the original results. Furthermore, isolates that had multiple resistance phenotypes and corresponding susceptible genotypes were retested for those drugs and the data were corrected if the phenotypic retesting differed from the original results. The AST results for each isolate are given in Data Set S1.

### DNA extraction.

Isolates were cultured on LJ medium at 37°C for 3 to 4 weeks at PHAS. To begin DNA extraction, two inoculation loops of bacteria were resuspended in 450 μl of TE buffer (10 mM Tris-HCl, 1 mM EDTA [pH 8.0]) and heated at 80°C for 20 min. The cells were freeze-thawed twice, and 30 μl of lysozyme (20 mg/ml) was added and incubated for 2 h at 37°C. Seventy microliters of 10% sodium dodecyl sulfate (SDS) and 5 μl of proteinase K (10 mg/ml) were added to the lysate, vortexed, and incubated for 10 min at 65°C, followed by addition of 100 μl of 5 mol/liter NaCl and 100 μl of 10% *N*-cetyl-*N*,*N*,*N*-trimethyl ammonium bromide. The tubes were vortexed until the solution was white and then incubated for 10 min at 65°C. DNA was extracted by two chloroform-isoamyl alcohol (24:1 [vol/vol]) treatments and precipitated by the addition of cold isopropanol, and the pellet was redissolved in TE buffer.

### Genome sequencing, variant analysis, and computational spoligotyping.

DNA samples were submitted from PHAS to the Broad Institute (Cambridge, MA) for whole-genome sequencing. For each sample, the DNA concentration was determined, and two sequencing libraries were created: a fragment library (∼180-bp inserts) and a jumping library (∼3- to 5-kb insert). Libraries were sequenced on Illumina HiSeq 2000 instruments generating 101-bp paired-end reads at ∼140× coverage. Reads from both libraries were aligned against the H37Rv reference genome (GenBank accession no. CP003248.2) using BWA (20), and variants were called using Pilon version 1.5 (21). Variant effects were predicted using the vcf annotator (https://sourceforge.net/projects/vcfannotator/). Genomic polymorphisms known to confer drug resistance in each strain were identified using a previously published list of polymorphisms based on current diagnostics, as well as variants implicated in resistance with experimental support in the literature (Table S1) (12). In a separate analysis, we identified all variants present in genes from Table S1, regardless of whether they were known to confer resistance or not. Finally, spoligotypes were computationally predicted by statistically testing for the presence of each of the 43 unique spacer sequences used in classical spoligotyping from sequence reads (22). The resulting profiles were matched to those at SITVITWEB (http://www.pasteur-guadeloupe.fr:8081/SITVIT_ONLINE/) to assign a spoligotype.

### Phylogenetic analysis.

An initial maximum clade compatibility Bayesian phylogenetic tree was constructed from the full complement of M. tuberculosis genomic SNPs using the program MrBayes version 3.2.4 (23). Support for individual clusters was quantified by clade posterior probabilities. To control for the influence of selection at loci involved in antibiotic resistance, the original set of genomic SNPs for these samples was filtered to remove SNPs from known antibiotic resistance loci. These loci were those identified as having a significant correlation with antibiotic resistance by two or more analytical methods in Farhat et al. (9). The remaining subset of sites from the M. tuberculosis genome was the resistance-neutral sequence data used for further phylogenetic analysis. A comparison of the full-genomic SNP Bayesian tree against the resistance-neutral Bayesian tree found no significant differences in the placement of well-supported clusters, so the resistance-neutral tree was used for further analysis. Evolutionary acquisitions of genotypic resistance were counted on the phylogenetic tree using the parsimony principle, and the resulting number was divided by the total number of phenotypically resistant isolates to determine the fraction of resistance acquired through *de novo* evolution.

### Accession number(s).

Short read data were submitted to NCBI under BioProject PRJNA200335.

## Supplementary Material

Supplemental material
