# Supplementary material for: Whole-Genome Sequencing of Mycobacterium tuberculosis Provides Insight into the Evolution and Genetic Composition of Drug-Resistant Tuberculosis in Belarus
Source: J Clin Microbiol. 2017 Jan 25;55(2):457–69. doi: 10.1128/JCM.02116-16 (PMC5277515; doi:10.1128/JCM.02116-16)
Supplement: Supplemental material [file supp_55_2_457__index.html]

Supplemental material 

# Whole-Genome Sequencing of Mycobacterium tuberculosis Provides Insight into the Evolution and Genetic Composition of Drug-Resistant Tuberculosis in Belarus

## Supplemental material

- Supplemental file 1 -

  Table S1 (Known resistance-conferring mutations)

  PDF, 85K
- Supplemental file 2 -

  Data Set S2 (Heat map of variants found at drug-resistance loci in all isolates)

  XLSX, 97K
- Supplemental file 3 -

  Data Set S1 (Patient metadata for the Belarus 2010-2013 isolates)

  XLSX, 63K
