# Supplementary material for: Whole-Genome Sequencing of Mycobacterium tuberculosis Provides Insight into the Evolution and Genetic Composition of Drug-Resistant Tuberculosis in Belarus
Source: J Clin Microbiol. 2017 Jan 25;55(2):457–69. doi: 10.1128/JCM.02116-16 (PMC5277515; doi:10.1128/JCM.02116-16)
Supplement: Supplemental material [file JCM.02116-16_zjm999095347s1.pdf]

Supplementary Table 1. Known resistance-conferring mutations. This list is based on current diagnostics and high confidence mutations from the literature as adapted from and described in Desjardins et al. (2016). Mutations with any non-match to the wild type probe in the Hain MTBDR*plus* or MTBDR*sl* were considered to confer resistance. *M. tuberculosis* codon numbering was utilized for all polymorphisms, including for polymorphisms within *rpoB*.

**Drug**	**Mutations**
Rifampicin	Any nonsynonymous mutation in the *rpoB* RRDR (430-562), inclusive of mutations on Hain MTBDR*plus* and GeneXpert
Isoniazid	*katG* S315<br>*inhA* promoter
Pyrazinamide	*pncA* promoter<br>*pncA* loss of function
Ethambutol	*embB* M306<br>*embB* G406<br>*embB* Q497
Streptomycin	*rrs* 513<br>*rrs* 516<br>*rrs* 906<br>*rrs* 907<br>*rpsL* K43<br>*rpsL* K88<br>*gidB* loss-of-function
Ofloxacin	*gyrA* G88<br>*gyrA* A90<br>*gyrA* S91<br>*gyrA* D94<br>*gyrB* QRDR (461-499)
Kanamycin	*rrs* 1400<br>*rrs* 1401<br>*rrs* 1483<br>*eis* promoter
Capreomycin	*rrs* 1400<br>*rrs* 1401

	rrs 1483<br>*tlyA* loss of function
Ethionamide	*inhA* promoter<br>*ethA* loss of function
Cycloserine	*ald* loss of function

References

Desjardins CA, Cohen KA, Munsamy V, Abeel T, Maharaj K, Walker BJ, Shea TP, Almeida DV, Manson AL, Salazar A, Padayatchi N, O'Donnell MR, Mlisana KP, Wortman J, Birren BW, Grosset J, Earl AM, Pym AS. 2016. Genomic and functional analyses of *Mycobacterium tuberculosis* strains implicate *ald* in D-cycloserine resistance. Nat Genet **48:**544-551.
